# Bioceramic hydroxyapatite-based scaffold with a porous structure using honeycomb as a natural polymeric Porogen for bone tissue engineering

**DOI:** 10.1186/s40824-021-00203-z

**Published:** 2021-01-19

**Authors:** Mona Sari, Puspa Hening, Chotimah, Ika Dewi Ana, Yusril Yusuf

**Affiliations:** 1 Department of physics, Faculty of Mathematics and Natural Science, Universitas Gadjah Mada, Yogyakarta, Indonesia; 2 Integrated Laboratory for Research and Testing, Universitas Gadjah Mada, Yogyakarta, Indonesia; 3 Department of Dental Biomedical Sciences, Faculty of Dentistry, Universitas Gadjah Mada, Yogyakarta, Indonesia

**Keywords:** Hydroxyapatite, Scaffold, Abalone mussel shells, Honeycomb, Bone tissue engineering

## Abstract

**Background:**

The application of bioceramic hydroxyapatite (HA) derived from materials high in calcium to tissue engineering has been of concern, namely scaffold. Scaffold pores allow for cell mobility metabolic processes, and delivery of oxygen and nutrients by blood vessel. Thus, pore architecture affects cell seeding efficiency, cell viability, migration, morphology, cell proliferation, cell differentiation, angiogenesis, mechanical strength of scaffolds, and, eventually, bone formation. Therefore, to improve the efficacy of bone regeneration, several important parameters of the pore architecture of scaffolds must be carefully controlled, including pore size, geometry, orientation, uniformity, interconnectivity, and porosity, which are interrelated and whose coordination affects the effectiveness of bone tissue engineering. The honeycomb (HCB) as natural polymeric porogen is used to pore forming agent of scaffolds. It is unique for fully interconnected and oriented pores of uniform size and high mechanical strength in the direction of the pores. The aim of this study was therefore to evaluate the effect of HCB concentration on macropore structure of the scaffolds.

**Methods:**

Bioceramic hydroxyapatite (HA) was synthesized from abalone mussel shells (*Halioitis asinina*) using a precipitation method, and HA-based scaffolds were fabricated with honeycomb (HCB) as the porogen agent. Pore structure engineering was successfully carried out using HCB at concentrations of 10, 20, and 30 wt%.

**Results:**

The Energy Dispersive X-Ray Spectroscopy (EDS) analysis revealed that the Ca/P molar ratio of HA was 1.67 (the stoichiometric ratio of HA). The Fourier Transform Infrared Spectroscopy (FTIR) spectra results for porous HA-based scaffolds and synthesized HA showed that no chemical decomposition occurred in the HA-based scaffold fabrication process. The porosity of the scaffold tended to increase when higher concentrations of HCB were added. XRD data show that the HCB was completely degraded from the scaffold material. The cell metabolic activity and morphology of the HA + HCB 30 wt% scaffold enable it to facilitate the attachment of MC3T3E1 cells on its surface.

**Conclusion:**

HCB 30 wt% is the best concentration to fabricate the scaffold corresponding to the criteria for pores structure, crystallographic properties, chemical decomposition process and cell viability for bone tissue engineering.

## Introduction

Calcium phosphate has excellent material properties in terms of biocompatibility and quality of integration with bone [1–4]. Bioceramic materials, such as hydroxyapatite (HA), which is from the calcium phosphate family, are alternative materials newly used in orthopedic applications because they can support bone tissue’s ability to regenerate itself. HA (Ca_10_(PO_4_)_6_(OH)_2_) is a major component of human bones and teeth and is commonly used in orthopedic, dental, and maxillofacial applications [5]. HA has the lattice parameters of *a* = 9.433 Å and *c* = 6.875 Å, and a variable Ca/P mol ratio of 1.67 [6–8]. The advantages of HA are its bioactivity, biocompatibility, and non-corrosiveness [6]. Since most of the mineral fraction in human bone tissue has the HA structure, HA can be effective in reconstructing human bone tissue [1].

A variety of techniques to synthesize hydroxyapatite have been developed, such as the sol-gel procedure [9, 10], precipitation from an aqueous solution [5–8, 11, 12] hydrothermal [13, 14] and solid-state reactions [15]. In this study, the precipitation method was selected to synthesize HA per several considerations. Majority of the synthesis approaches of HA does not require any organic solvent making it low cost process. This is a simple process with high throughput (87%), making the method suitable for large-scale (i.e., industrial) production.

HA made by chemical synthesis is called synthetic HA. Synthetic HA can be obtained from either synthetic or natural calcium rich sources. Some such natural materials include cow bones, fish bones, cuttlefish, and mussel shells [6]. In this study, abalone mussel shells (*Haliotis asinina*) from Indonesia are used as the natural compound for chemical synthesis, which are 90–95% calcium carbonate [16].

The application of HA derived from materials high in calcium to tissue engineering has been of concern, namely regarding the replication and reconstruction of artificial bone for various applications (such as scaffolds) [17]. Composite scaffold materials have a three-dimensional porous structure and large surface area and are biodegradable and biocompatible, which are major factors for cell growth and proliferation [18]. Many strategies to fabricate bone scaffolds have been developed including particulate leaching [19], gas foaming [20], phase separation [21], sponge templating [22], and polymeric porogen [1, 2]. The porogen leaching method is commonly used because the advantage of this simple method will produce traces of evaporated porogen particles in the form of pores on the scaffold. Furthermore, this method had the fabrication efficiency and the wide variety of porogens available [1].

Based on the previous study, the honeycomb (HCB) is unique for its fully interconnected and oriented pores of uniform size and high mechanical strength in the direction of the pores [23]. The HCB architecture is characterized by orderly unidirectional macropores, meaning channels that penetrate the materials. Moreover, as natural polymeric porogen, HCB structures offered high strength with low weight and less material [24]. Therefore, the use of HCB had the potential as a non-toxic pore forming agent of scaffold with a simple fabrication technique.

Scaffold pores allow for cell mobility and metabolic processes. Therefore, to improve the efficacy of bone regeneration, several important parameters of scaffold’s pore architecture must be carefully developed, including volume porosity [25]. Macroporous scaffolds have generally been fabricated by first mixing appropriate amounts of transient porogens with powders, and then evaporating, burning out, or dissolving the porogen or spacer [25]. Moreover, microporous structure played an important role in cell growth and differentiation of scaffold [23].

This work explores the potency of abalone mussel shells (*Haliotis* asinine) as the calcium source in HA synthesis. In this study, HA is synthesized via the precipitation method to obtain calcium oxide (CaO). The characteristics of this HA are then observed, including its effect on crystallinity, the mol ratio of Ca/P, and its thermal properties, as well as the functional groups of HA samples. HCB porogen at concentrations of 10, 20, and 30 wt% was used for the scaffold fabrication process. The physicochemical properties of the scaffold were characterized using Scanning Electron Microscopy-Energy Dispersive X-Ray Spectroscopy (SEM-EDS), X-Ray Diffraction (XRD), and Fourier Transform Infrared Spectroscopy (FTIR). The cell metabolic activity of the scaffold was determined through MTT assay.

## Materials and experimental methods

**Fig. 1:**
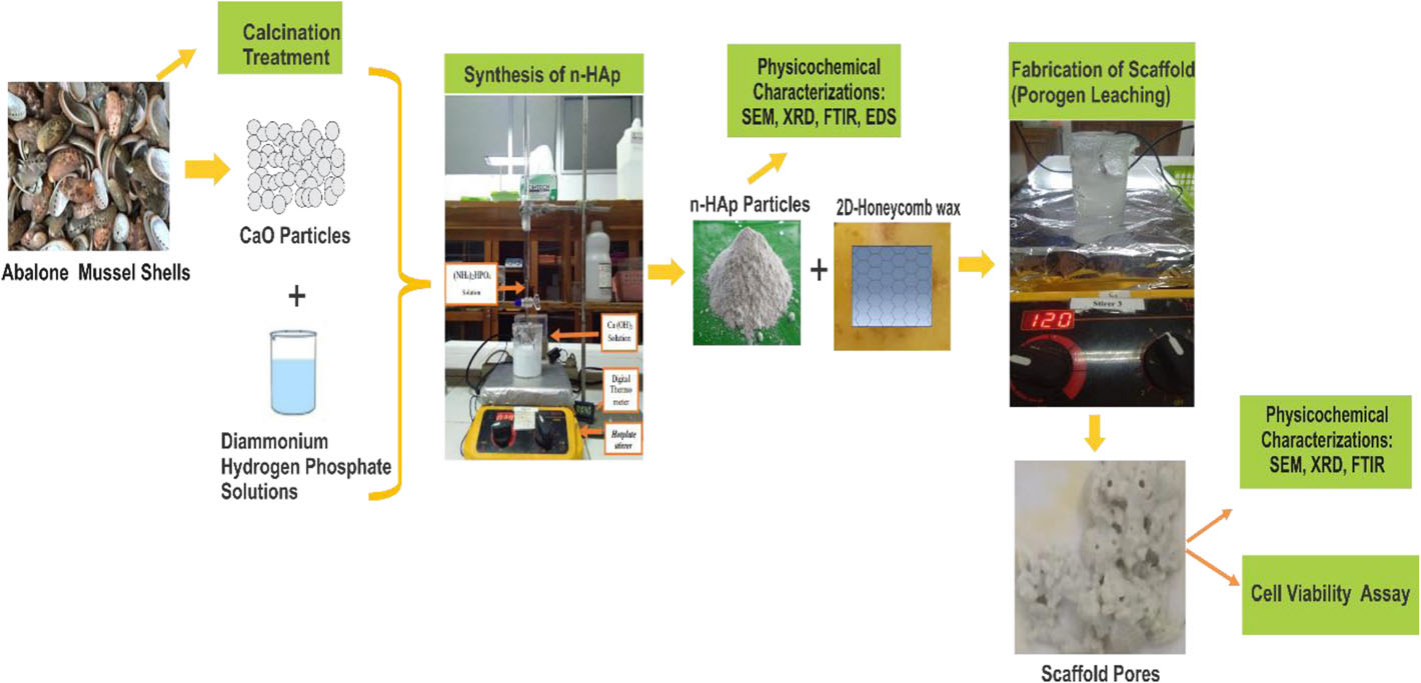
Schematic of methods to fabricate and characterize of nano-HA and scaffolds HA + HCB

The fabrication is divided into three main stages: preparation of calcium oxide (CaO) from abalone mussel shells, synthesis and characterization of nano-HA, and fabrication and characterization of scaffolds by varying HCB concentration at 10, 20, and 30 wt %. The schematic methods for this study were shown in Fig. 1.

### Materials

The abalone mussel shells used as a source of calcium carbonate (CaCO_3_) were taken from Bali, Indonesia. The precursors of diammonium hydrogen phosphate ([NH4]_2_ HPO_4_), ammonium bicarbonate (NH_4_HCO_3_), and ammonium hydroxide (NH4OH) 25% solution were purchased from Merck (USA). HCB was purchased from Sentra Madu Yogyakarta, Indonesia. Fetal bovine serum (FBS) and phosphate buffered saline (PBS) were purchased from Sigma-Aldrich (USA). Penicillin-streptomycin, fungizone, and MEM- *α* medium were purchased from Gibco (USA), (3-[4,5-Dimethylthiazol-2-yl]-2,5-diphenyltetrazolium bromide) (MTT) was purchased from Biobasic (USA), and dimethyl sulfoxide (DMSO) was purchased from Merck KGaA (Germany).

### Preparation of calcium oxide (CaO) from abalone mussel shells and synthesis of HA

The abalone mussel shells (*Haliotis asinina*) were cleaned in boiling water for 30 min and then washed using distilled water to remove attached materials, such as shell meat and algae. They were dried in an oven at a temperature of 100 °*C* for 6 h. A ball mill was used to reduce the shells to a smaller particle size. The powder from the shells was characterized using FTIR. It was then weighed and calcined using a furnace at a temperature of 1000 °C for 6 h to obtain the calcium oxide powder. It was then sieved using a 230mesh sieve to obtain more homogeneous particles.

An 2.5 g amount of calcium oxide was mixed with 50 ml of distilled water. Then, an (NH_4_)_2_ HPO_4_ solution (3.5323 g in 50 ml distilled water) was slowly added dropwise at a rate of 1 ml/min to the calcium oxide powder. The liquid mixture was stirred at a velocity of 300 rpm for 60 min at a temperature of 70 °C. The pH of the mixture was kept above 9 by adding ammonium hydroxide (NH_4_OH, 25%) 3 M. The mixture was then stirred by a magnetic stirrer for 50 min at 70 °C. The solution was subjected to an aging treatment for 24 h and washed using distilled water. The solution was filtered to obtain the precipitate of HA, which was dried at a temperature of 100 °C for 6 h. Finally, the HA was calcined at 1000 °C for 6 h using a furnace to obtain the pure HA.

### Preparation of wax from HCB

In this study, HCB wax was used in applying the porogen leaching method to synthesize porous HA-based scaffolds derived from abalone mussel shells. The wax was taken from the HCB by boiling it at a temperature of 70 °*C* for 10 min, and the wax was then filtered and cooled at a temperature of 25 °*C*.

### Fabrication of porous HA-based scaffolds

Scaffolds were produced by varying the HCB wax concentration of 10, 20, and 30 wt % to obtain different pore structures. The wax from HCB was added to the HA solution and sonicated at a temperature of 60 °*C* for 15 min. The solution was subjected to an aging treatment for 24 h. The solution was then stirred at a temperature of 60 °*C* and a velocity of 300 rpm until it turned into a gel. The gel was transferred to a crucible and heated at a temperature of 110 °C for 5 h. The HCB was leached from the scaffolds while the HA was densified and recrystallized during the sintering process at 900 °C for 2 h.

### Characterization of the synthesized HA and HA-based honeycomb scaffolds

#### Morphology, particle size distribution and porous structure analysis

The morphology of abalone mussel shells, calcinated abalone mussel shells, synthesized HA, and HA-based HCB scaffolds were observed by Scanning Electron Microscopy (SEM, Joel JSM-6510LA-1400, Japan). The pore size and percentage of porosity at least six macropores and twelve micropores of HA-based HCB scaffolds were measured using ImageJ software.

#### Composition of HA powders

Energy Dispersive X-Ray Spectroscopy (EDS), included in the SEM performed, was used to determine the composition of abalone mussel shells, calcinated abalone mussel shells, and synthesized HA. The carbon, calcium and phosphorus composition of HA powders were observed by EDS, and these results were used to calculate the mol ratio of Ca/P in HA powders.

#### Crystallography analysis

The crystallographic properties of abalone mussel shells, calcinated abalone mussel shells, synthesized HA, and HA-based HCB scaffolds were determined by XRD (PAN analytical Type X’Pert Pro, Japan). The XRD data were recorded in the range 2*θ* : 10 − 80° using Cu − Kα radiation at *λ* = 0.154 nm.

#### FTIR analysis

FTIR (Thermo Nicolet iS10, Japan) was conducted to determine the functional groups of the abalone mussel shells, calcinated abalone mussel shells, synthesized HA, and HA-based HCB scaffolds. Separately, the powder and then scaffold were ground and mixed with potassium bromide (KBr) and then passed into compact tablets [26]. The FTIR instrument was operated in the range of 400–4000 cm^−1^.

### Cell viability assay of the HA-based honeycomb scaffolds

#### Extraction solution of scaffold

An amount of 0.094 g HA-based HCB 30 wt% was mixed with 37.6 mL of distilled water for analysis to get a concentration at 2500 μg/ml. The solution was then stirred at a temperature of 60 °*C* at a velocity of 350 rpm until it turned into a homogeneous solution. It sonicated at a temperature of 60 °*C* for 1 h before the HA-based HCB 30 wt% scaffolds solution stored in the refrigerator.

#### Cell culture and seeding

Mouse osteoblast cells (MC3T3E1) were cultured in MEM- *α* medium (Gibco, USA) + 10% FBS (Gibco, CA, USA) + 2% Penicillin-Streptomycin (Gibco, CA, USA) + 0.5% Fungizone (Gibco, CA, USA). Prior to cell seeding, the HA-based HCB 30 wt% scaffolds solution was stored in the refrigerator. The cells were seeded on the bottom of a 96-well plate at a density of 2 x 10^4^ cells/well. The cell was incubated at 37 °*C* in 5% CO_2_ for 24 h. 100 μL amount of scaffold solution was added to the cells. The cell seeded on the scaffold was incubated at 37 °*C* in 5% CO_2_ for 24 h and 48 h.

#### MTT assay

Cell viability was studied by MTT assay for an incubation period of 24 h and 48 h. The measurement was taken for the HA + HCB 30 wt% scaffold and a control (the well without scaffold). The HA + HCB 30 wt% scaffold had the best results in terms of physicochemical properties, so it was used in the cell viability assay. In summary, the medium was discarded, 100 μL of MTT solution with a concentration of 0.5 mg/ml was added to the well, and it was incubated for 4 h. Then, DMSO was added to the well at 100 μL /well. The absorbance was recorded by Tecan Spark® (Tecan Trading AG, Switzerland) at 570 nm [27]. The cell viability was calculated by the following equation:

2.1 $$ Cell\ Viability\ \left(\%\right)=\frac{absorbance\ of\ scaffold- absorbance\ of\ control\ media}{absorbance\ of\ control- absorbance\ of\ control\ media}\ x\ 100 $$

Based on the Eq. (2.1), cell viability was determined according to the absorption value of the test cultures, expressed as a percentage of absorption for unstimulated control cultures [27]. Then, the IC_50_ value was analyzed out by non-linear regression.

### Statistical analysis

All MTT assay data were presented as means ± standard deviation (SD) and one-way analysis of variance (ANOVA) was used to analyze the obtained results followed by Tukey’s test. *P* values < 0.05 were considered statistically significant.

## Results

### HA synthesis from abalone mussel shells

**Fig. 2:**
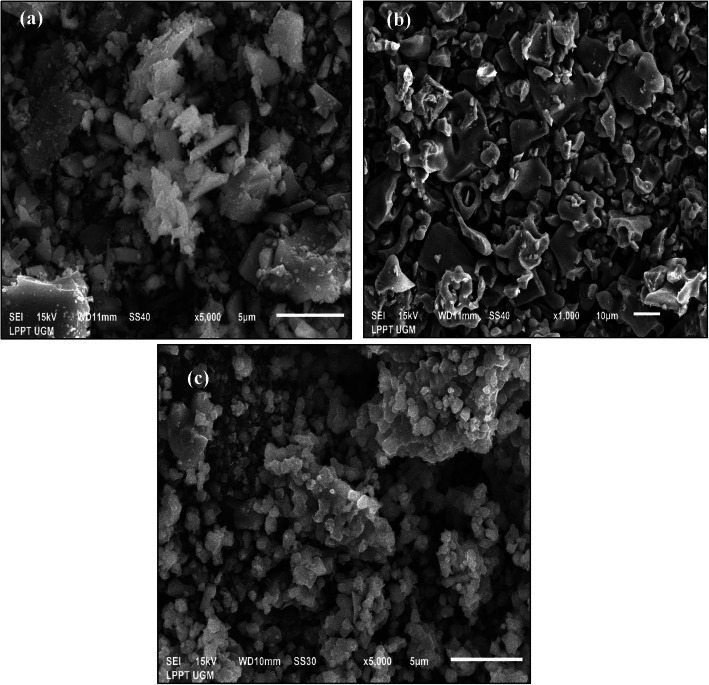
Morphology of **(a)** abalone mussel shells, **(b)** calcinated abalone mussel shells, and **(c)** synthesized HA

The morphologies and the composition of calcined mussel shells as well as the synthesized HA were probed under SEM and EDS analysis, respectively. In a previous study, the abalone mussel shells had a large particle shape with heterogeneous particle distribution, as shown in Fig. 2a. Figure 2b shows that the shells calcined at 1000 °*C* had an orderly shape with a more homogeneous and uniform distribution of particles. As shown in Fig. 2c, the synthesized HA had a small agglomerate shape and solid structure. The Ca levels of abalone mussel shells and shells calcined at 1000 °C were 37.47 and 61.33%, respectively.

**Fig. 3:**
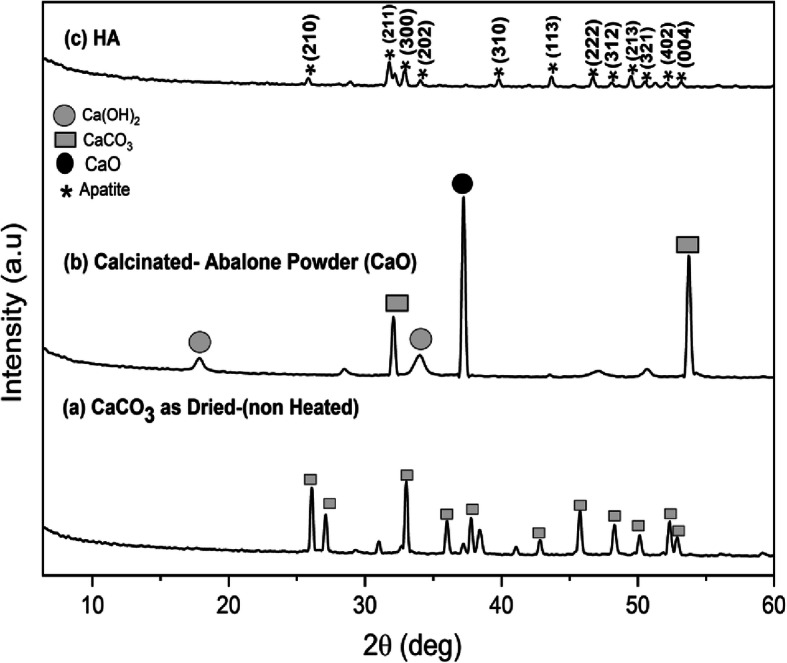
XRD pattern of **(a)** abalone mussel shells, **(b)** calcinated abalone mussel shells, and **(c)** synthesized HA

XRD characterization was used to determine the crystallography properties of the samples. As shown in Fig. 3a-b, the abalone mussel shells and the shells calcined at 1000 °C exhibited diffraction angles (2θ) of 33 ° and 37.21 °, respectively. The abalone mussel shells and the shells calcined at 1000 °*C* exhibited crystallite sizes of 48.18 ± 3.54 nm and 48.74 ± 3.25 nm, respectively. Their microstrains were 0.00253 and 0.00223, respectively. The XRD pattern of the HA made from abalone mussel shells is shown in Fig. 3c. The synthesized HA peaked at 31.76^o^ with an *hkl* index close to 211. These results agreed with data from the Joint Crystal Powder Diffraction Standard (JCPDS) No.09–0432. The crystallite size, microstrain, and X-ray density of the synthesized HA were (33.91 ± 7.5) nm, 0.00373, and 10.46 g/cm^3^, respectively.

**Fig. 4:**
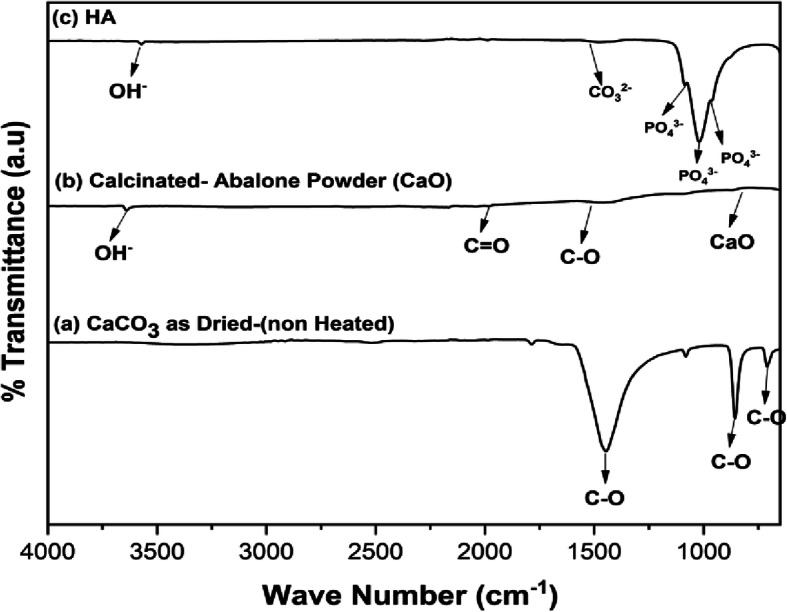
FTIR spectra of **(a)** abalone mussel shells, **(b)** calcinated abalone mussel shells, and **(c)** synthesized HA

FTIR spectra analysis was performed to identify the functional groups of samples themselves. As shown in Fig. 4a-b, the non-calcined abalone mussel shells did not display the OH^−^ stretching mode, the bond functional group of C = O and CaO. The CaO bond functional groups, the C = O bond, and the OH^−^ stretching functional groups were present in the abalone mussel shells at 1000 °*C*. The functional groups of CaO, C −O, C = O, and OH^−^ were observed at 873.88 cm^- 1^ and 1470.39 cm^− 1^, the C = O bond at 1792.50 cm^− 1^ and 2043.34 cm^− 1^_,_and the functional group of OH^−^ at 3640 cm^− 1^. As shown in Fig. 4c, the synthesized HA exhibited the functional group of HA. The HA exhibited the stretching mode of OH^−^ at 3571.66 cm^− 1^ and the bending modes of stretching _v_(P − O) mode of PO_4_^3−^ at 963.28, 1020.31, and 1085.81 cm^− 1^. HA exhibited the functional group of CO_3_^2−^ only at 1476.66 cm^− 1^.

### HA-based honeycomb scaffold

**Fig. 5:**
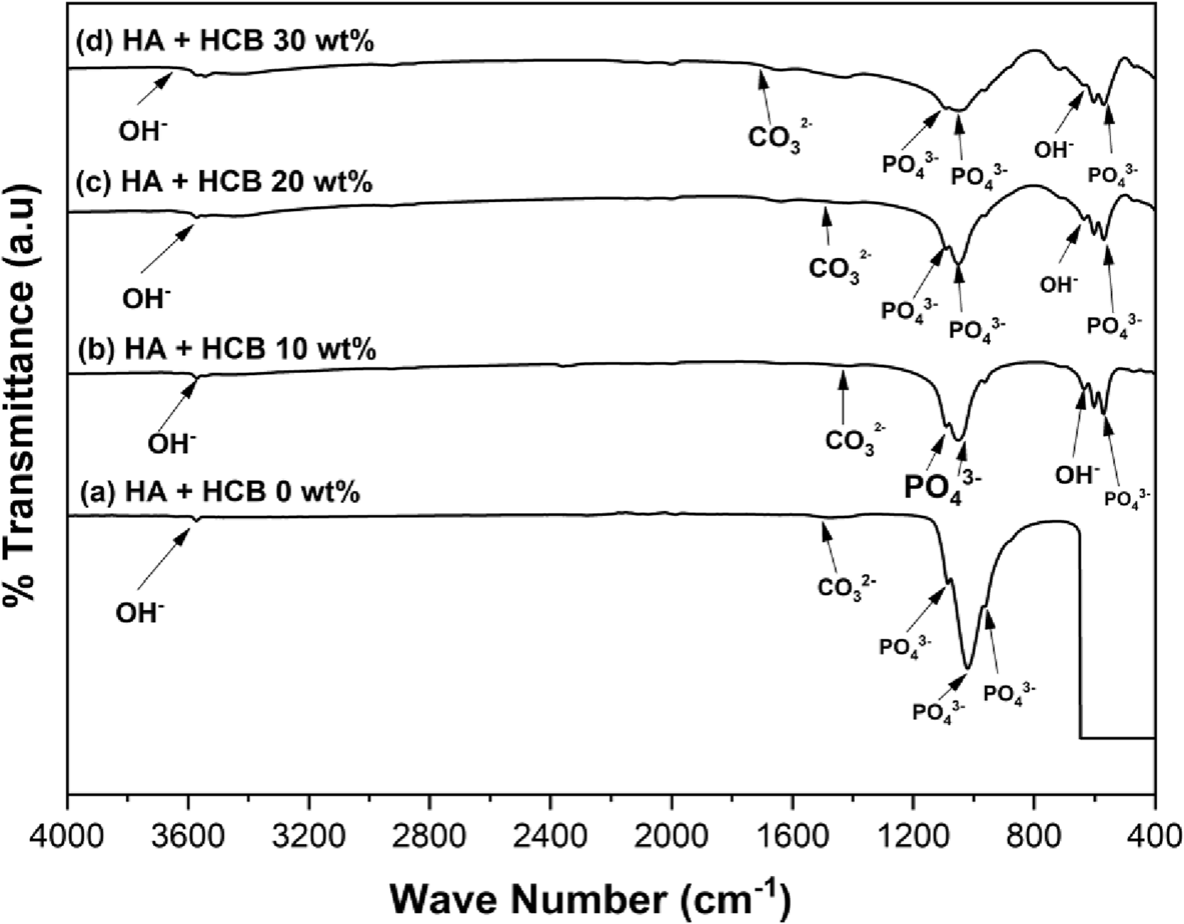
FTIR spectra of (**a**) HA + HCB 0 **wt** %, (**b**) HA + HCB 10 **wt** %, (**c**) HA + HCB 20 **wt** %, and (**d**) HA + HCB 30 **wt** %

The FTIR spectra data (Fig. 5) show that HA without HCB the functional groups of B-type CO_3_^2−^ at 1476.66 cm^− 1^, PO_4_^3−^ absorption at 963.28, 1020.31, and 1085.81 cm^− 1^ and the absorption band attributed to hydroxyl at 3571.66 cm^− 1^. PO_4_^3−^ absorption was observed at 602–570 cm^− 1^ and 1091–963 cm^− 1^ for all concentrations of HCB. For all variations in HA-HCB treatments, the absorption band attributed to hydroxyl was observed within the ranges of 636–635 cm^− 1^ and 3570–3543 cm^− 1^.

**Fig. 6:**
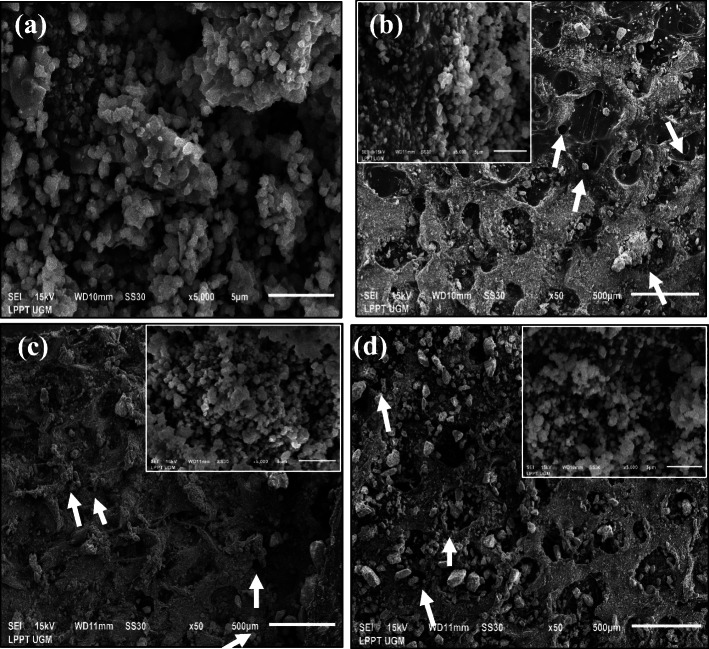
Morphology and porous structure of **(a)** HA + HCB 0 wt% (no pores), **(b)** HA + HCB 10 wt%, **(c)** HA+ HCB 20 wt%, and **(d)** HA + HCB 30 wt % (The white arrows indicated the HCB induces macropores)

According to the SEM results and Table 1, HA without HCB did not form the macropore and micropore structures (Fig. 6a). Micropore structure in pore size below 2 μm was shown by the insets of Fig. 6. As shown by the analysis of the SEM results using ImageJ software, the morphology of fabricated scaffolds formed bulk microporous. The addition of HCB 10 wt% resulted in the formation of pore structures on the scaffold (Fig. 6b). With the addition of HCB 20 wt%, the pore size decreased, and the structure became non-uniform (Fig. 6c). With the addition of HCB 30 wt%*,* it also decreased, but the structure became uniform (Fig. 6d).

**Table 1:** Pores size of scaffolds

No	Scaffold with concentrations variation	Macropore size (μm)	Micropore size (μm)
1	HA + HCB 0 wt%	No pore	No pore
2	HA + HCB 10 wt%	24.12 ± 2.07	0.36 ± 0.01
3	HA + HCB 20 wt%	11.47 ± 2.98	0.22 ± 0.001
4	HA + HCB 30 wt%	24.53 ± 2.11	1.03 ± 0.08

**Fig. 7:**
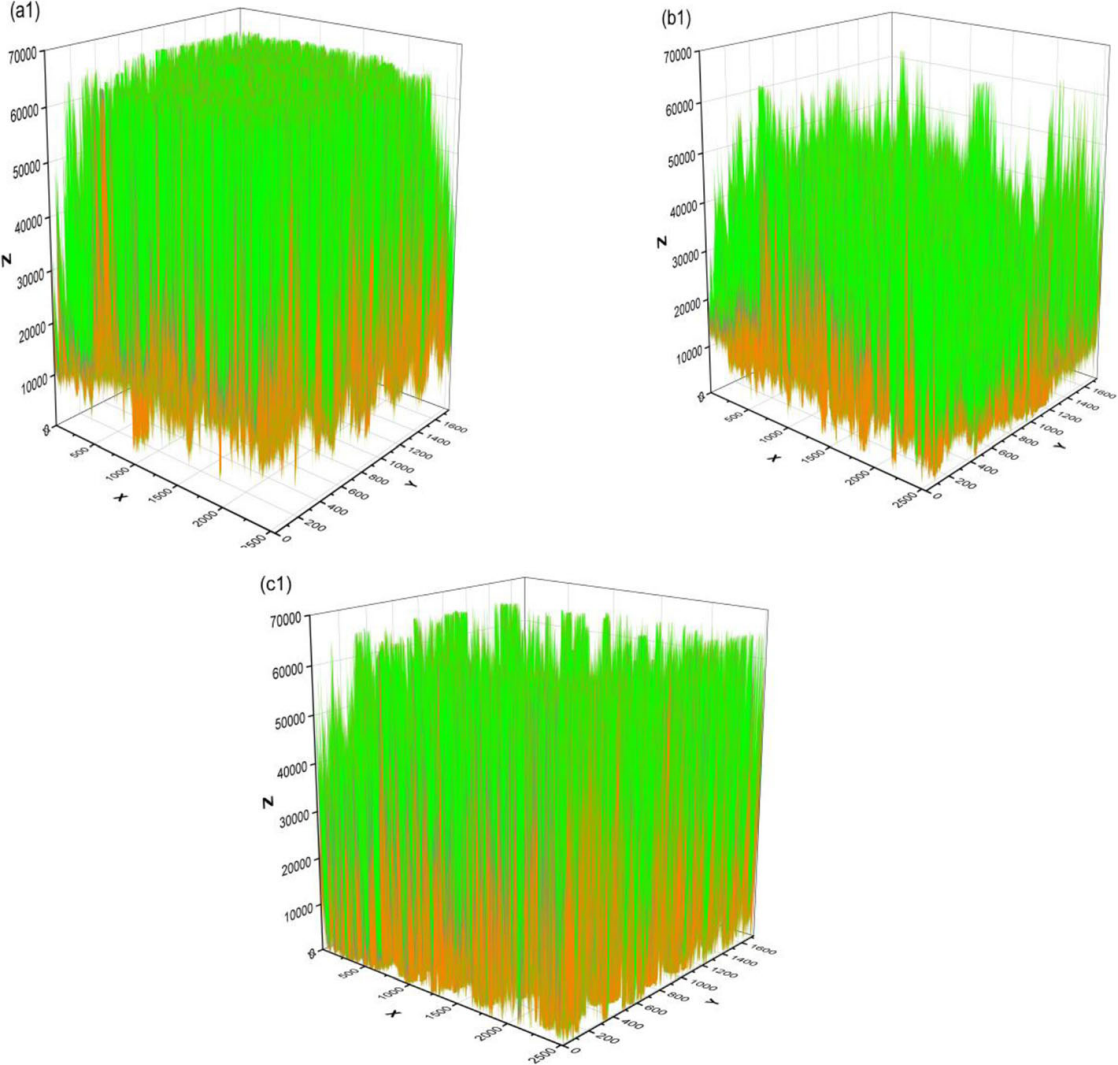
3D graphics and porosity of **(a)** HA + HCB 10 wt%, **(b)** HA + HCB 20 wt%, and **(c)** HA + HCB 30 wt%

Plotting 3D graphics and porosity was done with analysis two-dimensional porosity (surface area) of the scaffold from SEM images using the Origin software. The percentage of porosity can be analysis with calculate some volume values, including solid volume, integral volume, and pore volume based on 3D graphics. The pore distribution and solid particles can be evaluated from the prediction of their color distribution. In Fig. 7, the pore distribution on the HA-based HCB scaffolds is shown in orange and the solid particles are shown in green. With the addition of HCB 10 wt% (Fig. 7a), the pore distribution was adequately uniform, as indicated by the orange color, and had a porosity of 54.89%. The porosity of the scaffold increased to 70.27% with the addition of HCB 20 wt%; the distribution of pores still non-uniform, but the solid particles still dominated (Fig. 7b). With the addition of HCB 30 wt%, the porosity decreased to 66.67%, the pore distribution was not uniform, and the distribution of solid particles decreased (Fig. 7c).

**Fig. 8:**
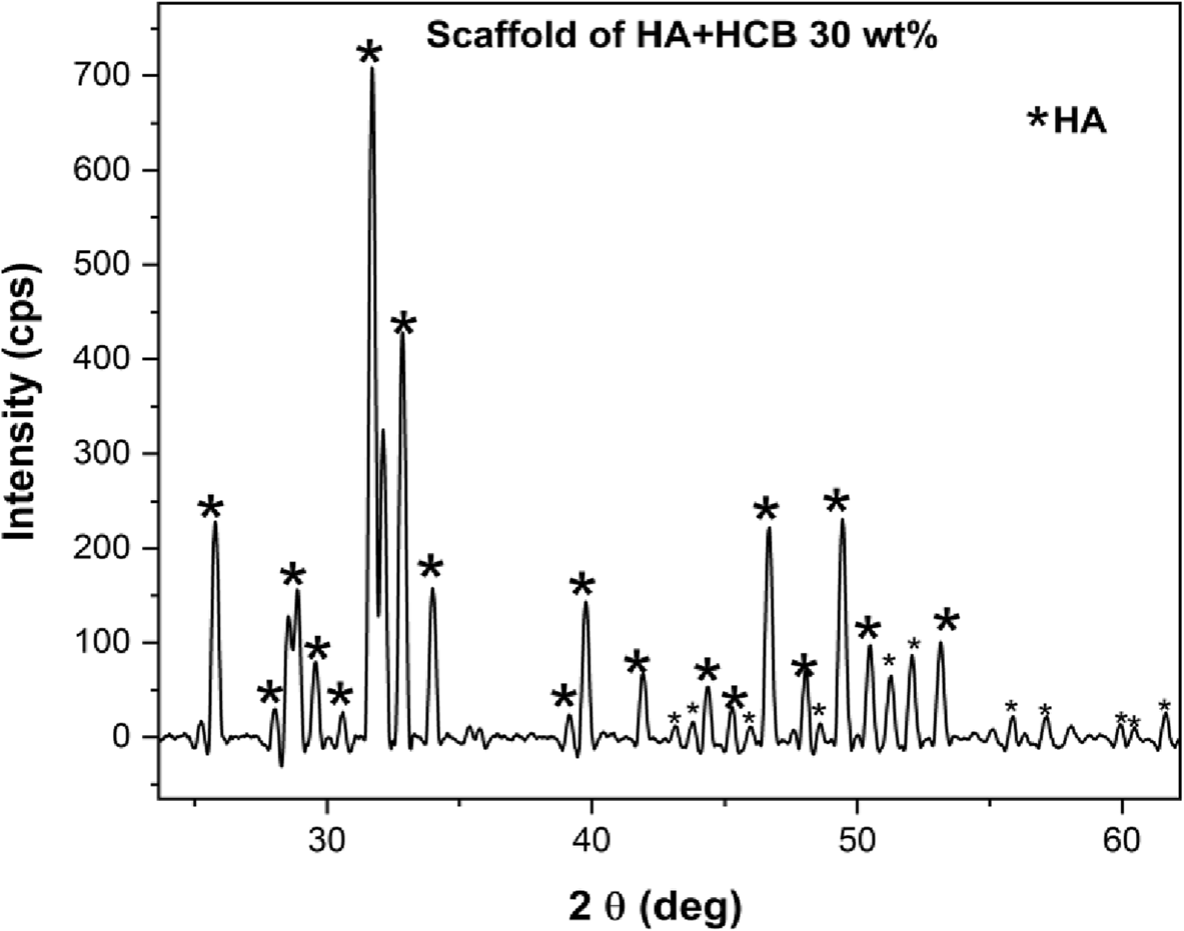
XRD pattern of scaffold HA + HCB 30 wt%

In this study, scaffold HA + HCB 30 wt% was demonstrated to be the best scaffold by its physicochemical characteristics. The crystallography analysis and cell metabolic activity studies carried out using XRD and MTT assay, respectively. The XRD pattern of the synthesized HA-based HCB scaffold was compared with JCPDS data 09–0432. The results of the XRD data (Fig. 8) show that the diffraction pattern formed was equivalent to the XRD pattern of HA. Using Scherrer equation, the crystallite size and microstrain of the scaffold HA + HCB 30 wt% were calculated to be (31.66 ± 2.00) nm and 0.00401, respectively.

**Fig. 9:**
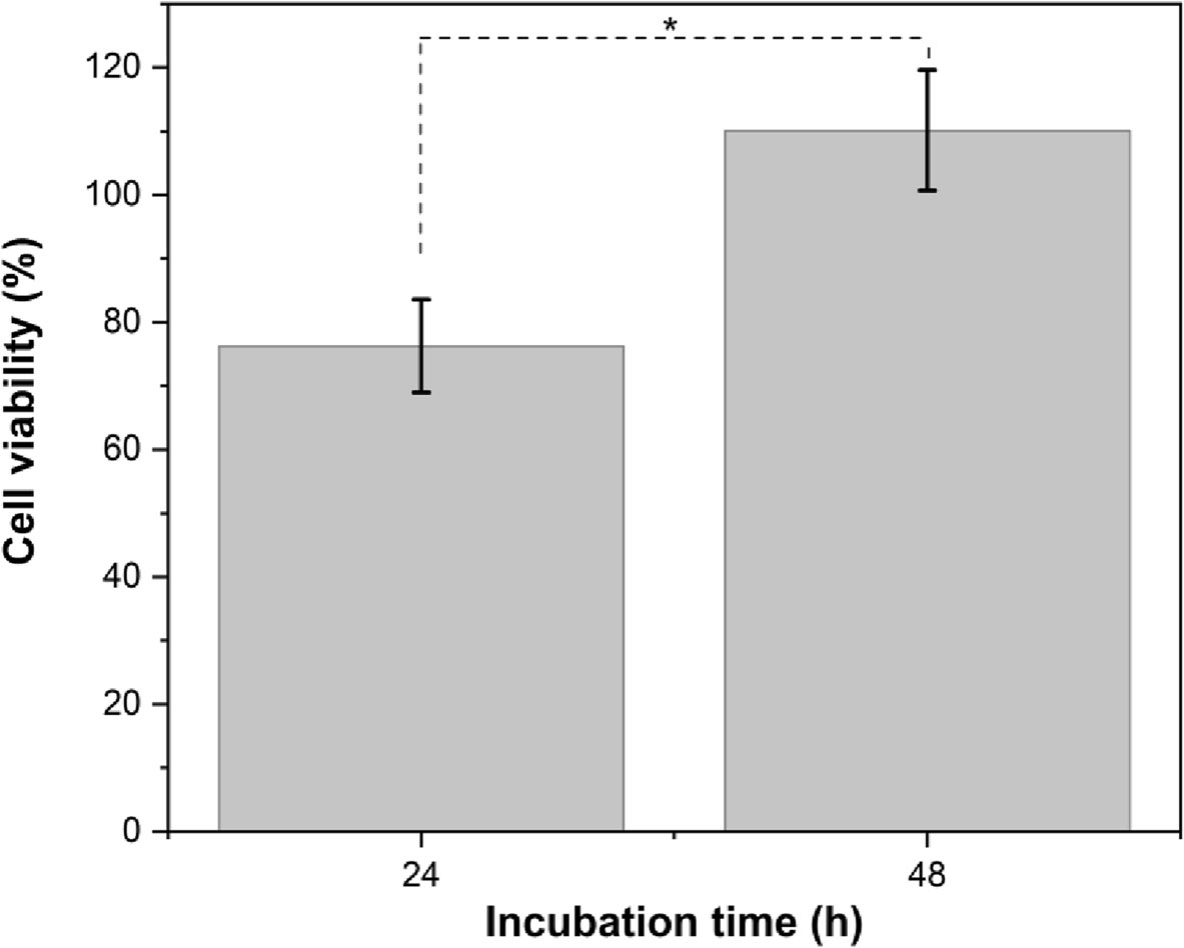
Cell metabolic activity of scaffold HA + HCB 30 wt% after being incubated 24 h and 48 h (*: *p* > 0.05)

Analysis of cell viability assay only comprehensively done for scaffold + HCB 30 wt% with incubation time of 24 h and 48 h. As shown in Table 1, only HA with addition of HCB 30 wt% has the potential as a scaffold in cell placement and cellular growth orientation because micropore size was ~1 μm. Generally, when the pore size of scaffold was 1–20 μm, it can be media for cellular growth [28]. The results of the MTT assay on scaffold HA + HCB 30 wt% showed that all serial doses of scaffold concentrations to MC3T3E1 cells were shown to be safe. As shown in Fig. 9, the cell viability after 24 h incubation indicated that scaffold HA + HCB 30 wt% viable for cells to attach because the cell viability of 76.26 ± 7.33%. The cell viability increased to 110.13 ± 9.44% after 48 h incubation. According to the one-way ANOVA to determine the effect of incubation times on the cell viability value, there was no significant difference in the average of cell viability value in two groups. These results were also supported by the analysis of the half maximal inhibitory concentration **(**IC_50_) value of MC3T3E1 cells in the HA + HCB 30 wt% scaffold with incubation times of 24 h and 48 h at 1276 and 1401 μg/ml, respectively. The IC_50_ is a quantitative measure that shows the amount of inhibitor substance needed to inhibit biological processes or biological components by 50%.

**Fig. 10:**
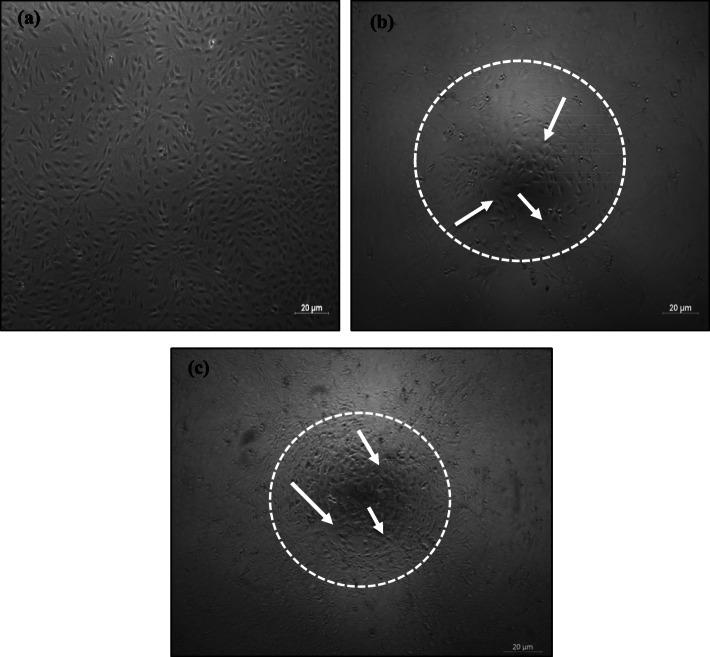
**(a)** MC3T3E1 cells’ morphology after sub-culture and Morphology on HA + HCB 30 wt% scaffold after being incubated for (b) 24 h, and (c) 48 h. (The white arrows indicated the MC3T3E1 cells grow in scaffold)

The MC3T3E1 cells mostly clustered and formed several sub confluent structures to ~ 80%, as shown in Fig. 10a. The nucleus of cells formed a round, and the cells were found to be well-connected the cell network [27]. The morphology of MC3T3E1 cells attached to the scaffold surface that had formed after 24 h and 48 h incubation is shown in Fig. 10b and c.

## Discussion

### HA synthesis from abalone mussel shells

In SEM images, the morphology of the abalone mussel shells differed considerably from that of the calcium oxide, indicating that the calcination process released CO_2_ and created cavities. The structure of the calcined shells aided the reaction with deionized water to hydrolyze the calcium oxide into csalcium hydroxide and form a solid suspension of calcium hydroxide. As shown in Fig. 2c, the morphology of synthesized HA resembled granules with uniform grains, but it had a rough surface. The SEM results showed that synthesizing HA using the precipitation method produced a fine grain of uniform size [6]. The EDS analysis shows increased the Ca concentration along with the calcination temperature treatment. Providing heat greatly helped the optimization of the decomposition reaction. When the CaCO_3_ compounds received the heat, the atoms moved faster; this movement broke the chemical bonds of CaCO_3_ into CaO and CO_2_. Increasing the calcination temperature inclined the breaking of the chemical bonds CaCO_3_ into CaO to occur faster. The synthesized HA exhibited a Ca/P molar ratio of 1.67 (the stoichiometric ratio of HA), so it was found to be consistent with natural HA.

The crystallographic properties of CaO powder and HA powders were determined by XRD. Based on the XRD pattern, CaO calcined at a temperature of 1000 °*C* had a large crystallite size with an increased crystallinity and a decreased amorphous phase level. In crystalline materials, it produced X-ray diffraction cleaner than the noise. This was due to the regular arrangement of atoms. CaO calcined at 1000 °*C* produced smaller microstrains compared to the abalone mussel shell. A small microstrain value indicated a small number of defects in the crystal [6]. The distance between the crystal planes of the HA was determined using the Scherrer equation to be 2.81 Å. This result is close to the crystal plane of the HA at 2.88 Å, making it appropriate by international standards (ISO 13779-3, ISO 13175-3) for HA implants [6, 29].

The existence of CO_3_^2−^ groups in the FTIR spectra were due to the reaction of calcium oxide with carbon dioxide in free air during synthesis. However, CO_3_^2−^ groups may have existed in the abalone mussel shells before the synthesis process. Carbon dioxide came into contact with the distilled water solvent in this reaction and released CO_3_^2−^ into the crystal lattice of the HA. The existence of CO_3_^2−^ groups is common because they occur naturally in human bones. It is unavoidable if HA synthesis is conducted in the open air. Therefore, there needs to be an environmental innertization (reactor) that passes inert gas, such as nitrogen (N_2_), so that the process of precursor mixing is free from outside air contamination [6].

### HA-based honeycomb scaffold

The FTIR spectra resulting from the porous HA-based scaffold and synthesized HA showed that no chemical decomposition occurred in the scaffold fabrication process. The FTIR spectra data (Fig. 5) show that HA with the addition of HCB at concentrations of 10, 20, and 30 wt % exhibited the functional groups of B-type CO_3_-HCB *v*_3_ vibration at 1424–1412 cm^− 1^. The infrared spectra were therefore used to determine whether the synthesized material showed the characteristic spectrum of HA.

According to the SEM analysis, the particles that evaporated from the porogen in the bioceramic and porogen mixture during the calcination process left the porous bioceramic scaffold morphology [2]. The results of macropore analysis found that the resulting macropore size was smaller than 100 μ m. However, several studies reported that the macropore size obtained was smaller than 100 μ m in several cases of polymeric scaffold [30, 31]. The separation of impurities from materials affects the boundary mobility of the particles. The porogen used in the HA scaffold fabrication process has the same effect as the impurity on the HA particles. In Fig. 6, the higher the concentration of the HCB added, the more the porosity of the scaffold tended to increase, but this was not the case with the HCB 30 wt%. The pores on the scaffold are expected to serve as the supply of nutrients to the bone tissue [1].

Based on the several previous research, the concentration of porogen on scaffold can affect pore size that is determined by several controlled parameter, including the presence of porogen [2], decomposition of residual porogen [32] and scaffold mass gain [33]. The HCB porogen in the suspension slowed down the densification process of the HA particles, causing the particle to be less interconnected by the higher porogen concentration in suspension. This cause more gaps between the HA particles and increases the porosity of the material [2]. In this study, macropore with smaller size (< 100 μ m) to the HCB concentration were assumed the result of gases released by the decomposition of residual HCB in scaffold [32]. Furthermore, decreasing porosity is influenced by scaffold mass gain [33], basically the mass percentage of HA in scaffold has been made equal (in this study, an amount of 0.5 g HA for HCB concentration at 10 wt%, 20 wt%, and 30 wt%, respectively). The increasing of porogen concentration affected micropore scaffold. It can fill the structure defects on one hand and strengthen the conjugation between HA grain size on the other [33]. Thus, the parameters of the HA and porogen composition needed to be improved for further study.

The XRD pattern of the synthesized HA-based HCB scaffold (Fig. 8) indicates that the HCB used was completely degraded from the scaffold material [1, 2]. For bone growth, the crystallinity must be lower because it causes dislocations, making it easier for cells to proliferate. Low crystallinity can be obtained by adding a carbonate substitution to HA. Thus, this parameter also needed to be improved for further study.

Cell viability is an essential aspect of determining a composite material ‘s potential for use in bone tissue engineering [26]. At least two factors should play a role in for the cell viability, such as grain size and chemical stability [34]. From the morphology of MC3T3E1 cells, it was evidenced that the scaffolds were cytocompatible towards the osteoblast cell line. The higher concentration of HA in the scaffolds likely brought more frequent clusters of MC3T3E1 cells comparable to results regarding cell metabolic activity.

## Conclusion

This study presents a successful synthesis of HA based on abalone mussel shells with a molar ratio of Ca/P 1.67 (the stoichiometric ratio of HA). Pore structure engineering using HCB at concentrations of 10, 20, and 30 wt% was successfully carried out. The FTIR spectra results for the porous HA-based scaffolds and synthesized HA showed that no chemical decomposition occurred in the HA-based scaffold fabrication process. The results of macropore analysis showed that the resulting macropore size was smaller than 100 μ m, so the parameters of the HA and porogen composition needed to be studied again; however, it was found that the higher the concentration of the HCB added, the more the porosity of the scaffold tended to increase. Overall the cell metabolic activity and morphology of the HA + HCB 30 wt% scaffold showed that it is able to facilitate the attachment of MC3T3E1 cells on its surface.

## Data Availability

The datasets used and/or analyzed during the current study are available from the corresponding author on reasonable request.

## Abbreviations

HA: Hydroxyapatite
HCB: Honeycomb
CaO: Calcium oxide
CaCO_3_: Calcium carbonate
OH^−^: Hydroxide ion
PO_4_^3−^: Phosphate ion
(NH_4_)_2_HPO_4_: Diammonium hydrogen phosphate
NH_4_HCO_3_: Ammonium bicarbonate
FBS: Fetal bovine serum
PBS: Phosphate buffered saline
MTT: 3-(4,5-Dimethylthiazol-2-yl)-2,5-diphenyltetrazolium bromide
DMSO: Dimethyl sulfoxide
SEM: Scanning electron microscopy
EDS: Energy dispersive X-ray spectroscopy
XRD: X-ray diffractometer
FTIR: Fourier transform infrared spectroscopy
IC_50_: Half maximal inhibitory concentration
